# Virginia State Dental Association

**Published:** 1870-12

**Authors:** 


### EDITORIAL DEPARTMENT.

*Virginia State Dental Association.*—It is with great pleasure we learn that the convention called by a notice which appeared in the October No. of this *Journal* was altogether successful, and that an Association has been formed on a sound basis which promises to do much good to the profession in that State. We feel a warm interest in this Association not only on account of Virginia being our native State, but for the reason that the prominent movers in this organization are friends, classmates and pupils; and aware of the energy and ability they possess, we feel assured that they will maintain and support it in a manner which will soon cause it to be recognized as one of the most prominent in the South.

We are indebted to the Secretary for the account of the proceedings of the first meeting, which appears in the present No. of the Journal.

